# Twelve-Month Follow-Up of a Randomized Controlled Trial of Internet-Based Guided Self-Help for Parents of Children on Cancer Treatment

**DOI:** 10.2196/jmir.6852

**Published:** 2017-07-27

**Authors:** Martin Cernvall, Per Carlbring, Anna Wikman, Lisa Ljungman, Gustaf Ljungman, Louise von Essen

**Affiliations:** ^1^ Clinical Psychology in Healthcare Department of Women’s and Children’s Health Uppsala University Uppsala Sweden; ^2^ Clinical Psychology Department of Psychology Stockholm University Stockholm Sweden; ^3^ Pediatric Oncology Department of Women’s and Children’s Health Uppsala University Uppsala Sweden

**Keywords:** cancer, parents, Internet, PTSS, depression, anxiety, clinical trial

## Abstract

**Background:**

A substantial proportion of parents of children on cancer treatment report psychological distress such as symptoms of post-traumatic stress (PTSS), depression, and anxiety. During their child’s treatment many parents also experience an economic burden.

**Objective:**

The aim of this study was to evaluate the long-term efficacy of Internet-based guided self-help for parents of children on cancer treatment.

**Methods:**

This study was a parallel randomized controlled trial comparing a 10-week Internet-based guided self-help program, including weekly support from a therapist via encrypted email, with a wait-list control condition. The intervention was based on cognitive behavior therapy (CBT) and focused on psychoeducation and skills to cope with difficult thoughts and feelings. Primary outcome was self-reported PTSS. Secondary outcomes were self-reported symptoms of depression, anxiety, health care consumption, and sick leave during the past month. Outcomes were assessed pre- and postintervention and at 12-month follow-up. Parents of children on cancer treatment were invited by health care personnel at pediatric oncology centers, and parents meeting the modified symptom criteria on the PCL-C were included in the study. Self-report assessments were provided on the Web.

**Results:**

A total of 58 parents of children on cancer treatment (median months since diagnosis=3) were included in the study (intervention n=31 and control n=27). A total of 18 participants completed the intervention, and 16 participants in each group participated in the 12-month follow-up. Intention-to-treat analyses revealed significant effects in favor of the intervention on the primary outcome PTSS, with large between-group effect sizes at postassessment (*d*=0.89; 95% CI 0.35-1.43) and at 12-month follow-up (*d*=0.78; 95% CI 0.25-1.32). Significant effects in favor of the intervention on the secondary outcomes depression and anxiety were also observed. However, there was no evidence for intervention efficacy on health care consumption or sick leave.

**Conclusions:**

Using the Internet to provide psychological interventions shows promise as an effective mode of delivery for parents reporting an increased level of PTSS and who consider Internet-based interventions as a viable option. Future research should corroborate these findings and also develop and evaluate interventions and policies that may help ameliorate the economic burden that parents may face during their child’s treatment for cancer.

## Introduction

Being a parent of a child diagnosed with cancer is burdensome. During the child’s treatment, parents have to cope with the disease itself, invasive treatments, and uncertainty about the child’s health and outcome. Indeed, parents of children on cancer treatment report psychological distress such as symptoms of post-traumatic stress disorder (PTSS) [1-3] and depression [4]. In addition, many parents of children on cancer treatment are affected economically. Cross-sectional studies indicate that the strain on household economy is highest during the first 6 months of treatment [5,6]. A recent longitudinal study found that the reduction in parents’ working hours is highest 2 months after the child’s diagnosis and then almost restored 1 year after end of treatment. However, 1 year after end of treatment, more mothers are still on sick leave than at the time of diagnosis [7]. Recent evidence from the same cohort suggests that reductions in working hours are restored 5 years after end of treatment [8].

There is evidence that psychological interventions can be of benefit to parents of children with chronic illnesses [9]. To the best of our knowledge, the intervention with best empirical support for reducing psychological distress among parents of children on cancer treatment is problem-solving training administered face to face [10,11]. This intervention is effective in reducing PTSS and depression among mothers of children on cancer treatment when compared with an inactive condition [11] and when compared with an active [12] condition consisting of nondirective support including active listening and reflective support. The intervention is 8 weeks in duration and has been evaluated up to 3 months after end of the intervention. A limitation of these studies is the lack of long term follow-up assessments. Importantly, there are no published findings regarding the effects of psychosocial interventions on health-related costs among parents of children on cancer treatment.

As the medical treatment for pediatric cancer is highly specialized, families where a child is treated for cancer often live far from the center where the child receives its care. This distance can make it difficult to maintain proper psychosocial and psychological support. Research has reported that less than half of parents who report a need to see a psychologist have had the opportunity to do so [13]. Cognitive behavior therapy (CBT) provided via the Internet is a promising treatment modality for a range of conditions [14], including parents of children with traumatic brain injury [15]. Providing interventions via the Internet could potentially increase access of support for parents of children who are receiving treatment for cancer. Recent developments and ongoing work in the wider field of interventions for parents of children with cancer also include Web-based CBT to improve quality of life in families of young cancer survivors [16].

We have developed a 10-week guided self-help intervention for parents of children on cancer treatment to be administrated via the Internet [17]. The intervention is based on principles from cognitive and behavioral therapies and aims to teach parents skills to cope with distress related to their child’s disease and treatment. We have previously reported that the intervention seems effective in the short term with significant reductions in the primary outcome PTSS and the secondary outcomes depression and anxiety, with large effect sizes at postassessment compared with a wait-list control condition [18]. The purpose of this study is to investigate the efficacy of the intervention including data from the controlled follow-up 12 months after randomization. PTSS was the primary outcome, and secondary outcomes included symptoms of depression, anxiety, and health-economic outcomes such as health care consumption and sick leave.

## Methods

### Design

This is a parallel randomized controlled trial including pre- and postassessments and a controlled follow-up 12 months after randomization, comparing an Internet-based guided self-help program with a wait-list control condition. Participants were recruited consecutively from five of the six Swedish pediatric oncology centers. Participants allocated to the intervention condition received the intervention immediately after randomization, whereas participants allocated to the wait-list condition received the intervention 12 months after randomization. Neither participants nor therapists in the study were blind to condition allocation. This study relied on self-reported outcomes.

### Participants and Procedure

Participants and procedure have been described in detail previously [18]. In brief, eligible participants were parents of children on treatment for a cancer disease who were fluent in Swedish, had access to a computer with an Internet connection, fulfilled the modified symptom criteria on the PTSD-Checklist-Civilian version (PCL-C) [19], a self-report instrument corresponding to the Diagnostic and Statistical Manual of Mental Disorders, 4th edition (DSM-IV) criteria for PTSD [20], and did not suffer from any other psychiatric disorder in immediate need of treatment. The modified symptom criteria comprise scoring ≥3 on at least one of five symptoms of reexperiencing, one of seven symptoms of avoidance and numbing, and one of five symptoms of hyperarousal, corresponding to partial PTSD [21]. A power analysis indicated that a total of 72 participants needed to be included to, with a power of .80, detect a large effect size (*d*=0.80) on the PCL-C assuming *P*<.05. Given that data on health care visits and sick leave were collected and that such variables generally vary more than clinical efficacy, a sample of 120 participants was estimated appropriate. However, the participation rate during the 4 years of inclusion was considerably lower than expected, and due to administrative reasons inclusion had to be terminated before this sample size was reached.

Potential participants were approached in person by a nurse or physician on the wards at the pediatric oncology centers 4-12 weeks after the child’s diagnosis. In the initial protocol, potential participants were to be approached 1-2 weeks after diagnosis. However, during the first months of inclusion it was evident that this was not feasible, and parents often were approached later, and the protocol was changed to the time frame reported. Potential participants were provided written and oral information about the study and were asked for written consent to participate. Nurses responsible for the recruitment at each center were not affiliated with the research group responsible for the study but were reimbursed for their work. Parents were informed that the intervention would be 10 weeks in duration and require approximately 4 hours of work per week to complete. A psychologist from the research group contacted consenting parents via telephone, and parents were instructed to complete the screening and preassessment on the Web. Thereafter, a clinical interview with a psychologist was conducted via telephone. Three master’s level psychologists conducted the interviews. Participants in the intervention condition completed the postassessment on the Web immediately after the intervention. Participants in the wait-list control condition completed the postassessment on the Web after the corresponding time (ie, 10-weeks post randomization). Participants in both conditions completed the follow-up on the Web 12 months after randomization. Thereafter, participants in the wait-list condition were offered access to the intervention. The procedure was approved by the regional ethics review board in Uppsala (Dnr 2008/238), and all participants provided written informed consent. Inclusion to the study started in April 2010. During the planning of this study, trial registration was less common in the field of psychology than it is currently. Therefore, this trial was not registered in a World Health Organization (WHO) accredited trial registry.

### Intervention

The intervention consists of Internet-based guided self-help provided during 10 weeks. The material has been described in detail [17] as well as its use in the current trial [18]. It consists of approximately 100 pages (A4 format) of text and visual material presented in nine modules. The intervention is based on CBT-principles [22-24] and focuses on psycho-education and teaching strategies to manage the current situation of being a parent of a child on cancer treatment and the stressors it entails. Components include relaxation training, coping with distressing thoughts and feelings, behavioral experiments, problem-solving, structured emotional writing, values and goal setting, general self-care, and maintenance of behavior change.

Participants accessed the intervention material via a Web-based portal and were instructed to work with each module for 1 week. Each participant was assigned a therapist and was instructed to send completed homework assignments via the portal to the therapist once each week. The therapist provided written feedback on each assignment and general progress through the intervention via the portal. The sequence of modules was fixed, which enhanced treatment integrity. If participants had not submitted their homework they were sent an email reminder to log in to the system. During the recruitment phase and in the informed consent, participants had been informed that the intervention would imply about 4 hours of work per week.

There were three therapists in the study. One licensed psychologist and two psychologists with a master’s degree in psychology. The two nonlicensed psychologists received supervision from the licensed psychologist. The therapists were affiliated with the research group responsible for the study and independent from centers from which participants were recruited. Logging of therapist time and activities was not supported by the portal, but therapists were instructed to spend 15-20 min per week when providing feedback to each participant. Individuals randomized to the intervention who had their partner included in the study received individual feedback but were encouraged to work together with their partner during the intervention if that suited them.

All participants were free to receive psychosocial services from the regular health care. These may have differed between centers as there are no standardized psychosocial services for parents within the Swedish pediatric oncology care setting.

### Outcomes

#### Primary Outcome

Post-traumatic stress symptoms related to the child’s cancer disease were assessed with the PCL-C [19]. The PCL-C consists of 17 items rated on a 5-point scale (1=not at all and 5=extremely), corresponding to the items assessing the B (reexperiencing), C (avoidance and numbing), and D (hyper-arousal) criteria in the DSM-IV. The instrument has adequate internal consistency, test-retest reliability, and evidence for convergent and discriminant validity when compared with other well-established measures of PTSS, depression, and general anxiety [25]. A score of 44 or above on the full scale suggests a diagnosis of PTSD [26]. Cronbach alpha in the current sample was .84 at preassessment.

#### Secondary Outcomes

Depression was assessed with the Beck Depression Inventory-II (BDI-II) [27] consisting of 21 items rated on a 4-point scale (0-3). The BDI-II has good concurrent validity with the BDI and the Hamilton Psychiatric Rating Scale; the suggested cut-offs are 0-13 indicating minimal, 14-19 mild, 20-28 moderate, and 29-63 severe depression. Cronbach alpha in the current sample was .82 at preassessment. Anxiety was assessed with the Beck Anxiety Inventory (BAI) [28] consisting of 21 items rated on a 4-point scale (0-3). The BAI has good test-retest reliability and convergent validity; suggested cut-offs are 0-7 indicating minimal,

8-15 mild, 16-23 moderate, and 24-63 severe anxiety. Cronbach alpha in the current sample was .87 at preassessment. Economic outcomes were assessed with questions from the Trimbos iMTA questionnaire for costs associated with psychiatric illness (TiC-P) [29]. Questions used in this report assessed frequency of health care use and sick leave during the last month. For health care use, one or more visits to different health care providers were coded as a “yes.” Similarly, for sick leave, 1 day or more of absence due to sickness during the last month was coded as a “yes.”

### Randomization

Randomization was performed by a consultant independent from the research group. Proc Plan SAS version 9.1 (SAS Institute, Cary, North Carolina, USA) was used to generate the randomization schedule, and sealed envelopes were prepared by the consultant and handed to the research group. Parents were randomized in 1:1 ratio to intervention or wait-list, and the randomization was stratified according to center, parental gender, and whether a participant had a partner in the study or not. Partners were randomized to the same condition.

### Statistical Analyses

Independent samples *t* test, Mann Whitney *U* test, chi-square test, and Fisher exact test were used to test for between-group differences on demographic characteristics and outcomes at preassessment. For the continuous outcomes (PCL-C, BDI-II, and BAI), mixed effects modeling with full maximum likelihood estimation was used to examine potential change across assessments and effects of the intervention [30], including random intercepts and slopes. Analyses were conducted on the intention-to-treat (ITT) principle where all randomized participants are included in the analyses, assuming missing data to be missing at random [31]. The data missing mechanism was assessed before the main analyses by exploring the relationships between characteristics at preassessment and missing data. Condition was dummy coded with intervention=1 and control=0. Assessment (pre, post, and 12-month follow-up) was included as a continuous time-variable coded as pre=0, post=1, and follow-up=2. Models were tested stepwise with increasing complexity and selected based on model fit indices, that is, -2loglikelhood difference test, Akaike Information Criteria (AIC), and Bayesian Information Criteria (BIC). All models tested included variance component as covariance structure. The complete model development is presented in Multimedia Appendices 1-3. For each outcome, we started with an unconditional model including a random intercept and slope (model A), as a second step we added linear growth as a fixed effect (model B), as a third step we added group as a fixed effect (model C), and as a fourth step we added quadratic growth as a fixed effect (model D). Standardized effect sizes (Cohen *d*) between groups at postassessment and 12-month follow-up were calculated using the model estimated mean differences (by recoding the continuous time-variable to −1 0 1 to set the intercept at the postassessment and −2 −1 0 to set the intercept at the 12-month follow-up assessment) and standard deviations from preassessment [32]. The magnitude of the effect expressed in *d* was interpreted according to Cohen [33], that is, 0.2=small, 0.5=medium, and 0.8=large. Variables pertaining to economic data consisted of frequencies of health care visits and days on sick leave. Due to the distribution of these frequencies, these variables were recoded to binary categorical variables representing no visits or days on sick leave (coded as 0) and any one or more visits or days on sick leave (coded as 1). However, ITT analyses with these data using, for example, generalized estimating equations, were not feasible due to the small sample size, and results for this secondary outcome are based on the available data excluding participants with missing data. Between-group differences at pre-, post- and follow-up assessments were analyzed with chi-square tests or Fisher exact test. Due to the small sample size, clustering by center and child was not addressed in any of the analyses. Analyses were performed in IBM SPSS Statistics 22 (IBM Corporation, Armonk, New York, USA).

## Results

### Recruitment and Baseline Characteristics

**Figure 1:**
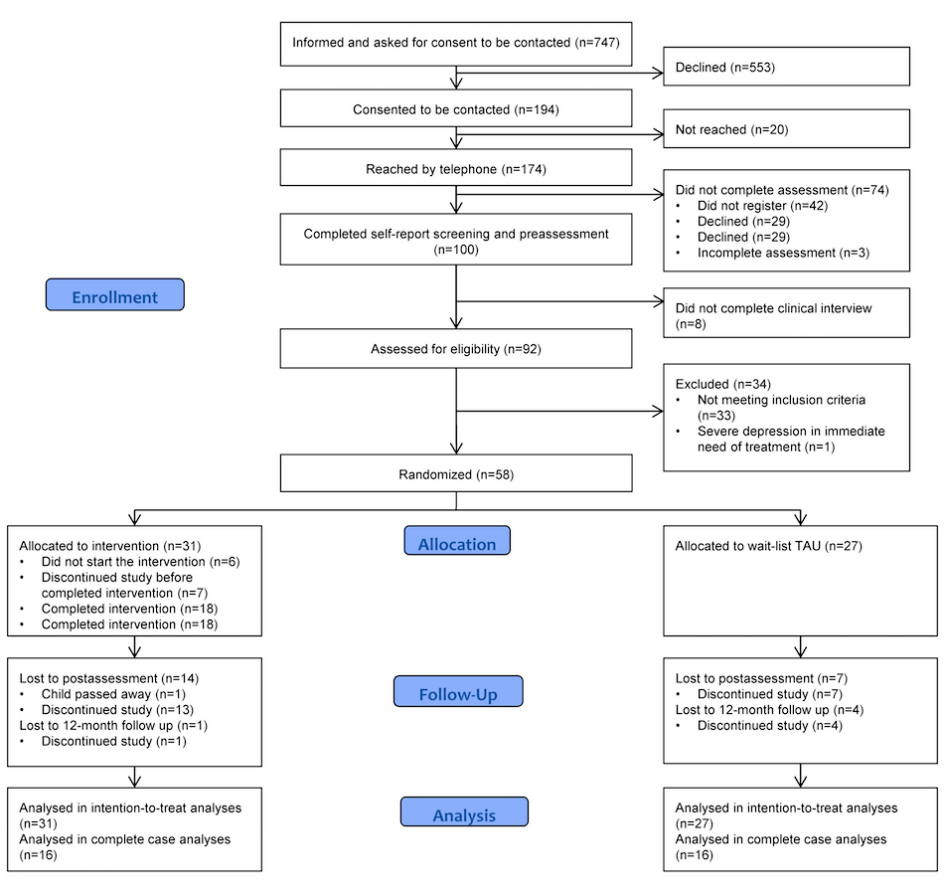
Consort diagram of participant flow through the study.

Participant flow through the study is outlined in Figure 1. From April 2010 to May 2014, 747 potential participants were informed about the study and asked for consent to be contacted again of which 553 declined. A total of 174 were reached by telephone, and 92 of these completed the screening, preassessment and clinical interview, and were assessed for eligibility. Fifty-eight parents of 46 children were included and randomized. Baseline characteristics have been described previously [18], and observed characteristics are presented in Tables 1 and 2. There were no differences in baseline characteristics between groups except for the BAI with a higher score in the intervention group. The last follow-up assessment took place in August 2015.

### Attrition and Adherence

Fourteen participants in the intervention group (45%, 14/31) and 7 in the wait-list group (26%, 7/27) did not provide postassessments. Furthermore, 1 participant in the intervention group and 4 in the wait-list group did not provide follow-up assessments, resulting in 16 participants in each group at 12-month follow-up. At preassessment, there were no differences in terms of demographic characteristics or outcome measures between those who provided post- and follow-up assessments and those who did not (*P* value ranging .14-.98) except for gender where fathers were less likely to provide data at 12-month follow-up compared with mothers (χ^2^_1_=6.4, *P*=.01).

As reported previously [18], adherence to the intervention was operationalized as the numbers of treatment modules accessed and log-ins to the Web-based portal. In the intervention group, 6 participants did not start the intervention, and 7 discontinued before completion. A total of 18 out of 31 participants were considered as completers, representing 58% of those allocated to the intervention. For the ITT-sample, the median number (interquartile range [IQR]) of accessed modules was 4 (4), and the median number (IQR) of log-ins was 13 (22). For the completer-sample, the median number (IQR) of accessed modules was 5 (3.5), and the median number (IQR) of log-ins was 20 (20).

**Table 1:** Baseline characteristics of parents.

Characteristics		Total sample n=58	Intervention n=31	Wait-list n=27	*P* value
Mothers, n (%)		39 (67)	21 (68)	18 (67)	.76
Partner also included in study, n (%)		22 (38)	12 (39)	10 (37)	.90
Age, mean (SD^a^)		38 (7.2)^b^	40 (7.4)^c^	36 (6.6)^d^	.06
Living with child’s biological parent, n (%)		51 (88)	26 (84)	25 (93)	.31
Completed university studies, n (%)		30 (52)	17 (55)	13 (48)	.61
In active employment, n (%)		47 (81)	26 (84)	21 (78)	.56
Median distance in km to POC^e^ (IQR^f^)		35 (61)	39 (62)	20 (50)	.39
Experience of previous traumatic event(s), n (%)		26 (45)	14 (45)	12 (44)	.96
Median months since child´s dx^g^ (IQR)		3.0 (3.0)^h^	3.0 (2.25)^i^	3.0 (3.0)	.39
**Outcomes, mean (SD)**					
	PCL-C^j^	49.1 (10.3)	51.5 (9.4)	46.6 (10.7)	.06
	BDI-II^k^	20.6 (7.5)	21.6 (8.1)	19.3 (6.7)	.24
	BAI^l^	14.7 (7.6)	17.2 (7.8)	11.9 (6.3)	<.01

^a^SD: standard deviation.

^b^n=55.

^c^n=29.

^d^n=26.

^e^POC: pediatric oncology center.

^f^IQR: interquartile range.

^g^dx: diagnosis.

^h^n=57.

^i^n=30.

^j^PCL-C: PTSD-Checklist-Civilian version.

^k^BDI-II: Beck Depression Inventory-II.

^l^BAI: Beck Anxiety Inventory.

**Table 2:** Baseline characteristics of children.

Characteristics		Total sample n=46	Intervention n=25	Wait-list n=21	*P* value
Female, n (%)		25 (54)	16 (64)	9 (43)	.15
Median age (IQR^a^)		5 (9.0)	6 (10.5)	4 (8.0)	.26
**Diagnosis, n (%)**					.33
	Leukemia	24 (52)	13 (52)	11 (52)	
	Sarcoma	8 (17)	5 (20)	3 (14)	
	Lymphoma	3 (7)	3 (12)	0 (0)	
	CNS^b^-tumor	7 (15)	2 (8)	5 (24)	
	Other malignant disease	4 (9)	2 (8)	2 (10)	

^a^IQR: interquartile range.

^b^CNS: central nervous system.

**Table 3:** Estimated outcomes of mixed effects models and effect sizes (n=58 in intention-to-treat analyses). Cohen *d* is the standardized mean difference and was calculated using the estimated mean difference and the standard deviation of the complete sample at the preassessment.

Outcome		Degrees of freedom	Estimate (SE^a^)	95% CI	*F* test value	*t* test value	*P* value	
**PCL-C^b^**							
	Intercept	1, 66.6	46.26 (2.09)	42.08-50.44	488.11	22.10	<.001
	Group	1, 66.6	5.22 (2.86)	−0.49 to 10.94	3.33	1.82	.07
	Linear	1, 58.0	−2.29 (4.06)	−10.42 to 5.84	0.32	−0.56	.57
	Quadratic	1, 52.1	1.11 (2.00)	−2.90 to 5.14	0.31	0.56	.58
	Linear × Group	1, 59.2	−21.87 (5.92)	−33.73 to −10.02	13.63	−3.70	<.001
	Quadratic × Group	1, 50.8	7.47 (2.90)	1.63-13.30	6.61	2.57	.01
	Difference POST		−9.16 (3.50)	−16.09 to −2.23	6.86		.01
	*d* POST		0.89	0.35-1.43			
	Difference 12mFU		−8.07 (3.67)	−15.33 to −0.8	4.84		.03
	*d* 12mFU		0.78	0.25-1.32			
**BDI-II^c^**							
	Intercept	1, 63.9	19.33 (1.45)	16.43-22.22	177.50	13.32	<.001
	Group	1, 64.2	1.62 (1.99]	−2.36 to 5.59	0.66	0.81	.42
	Linear	1, 37.5	0.41 (0.92)	−1.47 to 2.28	0.19	0.44	.66
	Quadratic		-	-			-
	Linear × Group	1, 36.1	−5.58 (1.31)	−8.23 to −2.92	18.12	−4.26	<.001
	Quadratic × Group		-	-			-
	Difference POST		−3.91 (1.89)	−7.68 to −0.13	4.29		.04
	*d* POST		0.52	−0.003 to 1.04			
	Difference 12mFU		−9.41 (2.49)	−14.34 to −4.48	14.31		<.001	
	*d* 12mFU		1.25	0.69-1.82			
**BAI^d^**							
	Intercept	1, 57.8	11.48 (1.52)	8.44-14.51	57.36	7.57	<.001
	Group	1, 57.9	5.11 (2.08)	0.95-9.26	6.06	2.5	.02
	Linear	1, 33.0	1.84 (0.83)	0.15-3.52	4.93	2.2	.03
	Quadratic		-	-			-
	Linear × Group	1, 32.2	−6.06 (1.17)	−8.44 to −3.67	26.80	−5.2	<.001
	Quadratic × Group		-	-			-
	Difference POST		0.94 (1.98)	−4.91 to 3.03	0.23		.23
	*d* POST		0.12	−0.39 to 0.64			
	Difference 12mFU		−6.99(2.50)	−11.94 to −2.04	7.85		.006
	*d* 12mFU		0.92	0.38-1.46			

^a^SE: standard error.

^b^PCL-C: PTSD-Checklist-Civilian version.

^c^BDI-II: Beck Depression Inventory-II.

^d^BAI: Beck Anxiety Inventory.

**Figure 2:**
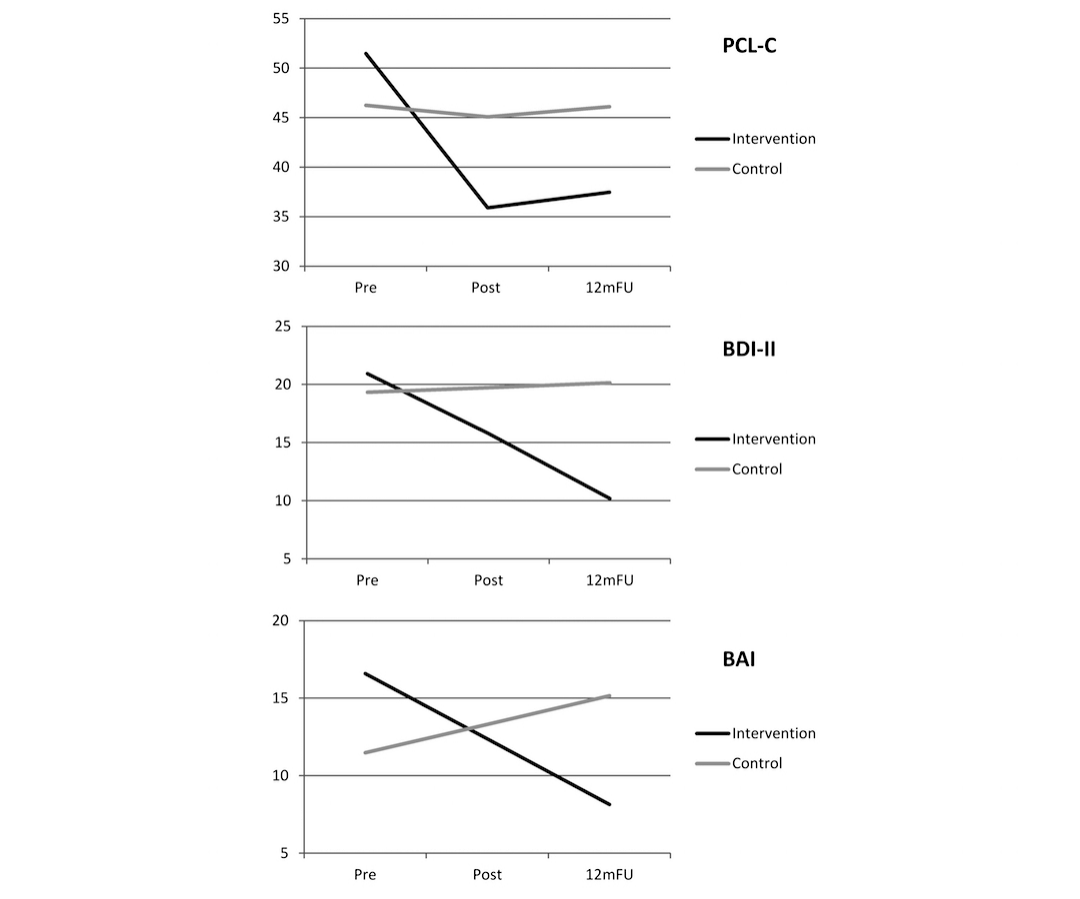
Estimated outcomes from mixed models with continuous variables.

### Outcomes

Table 3 and Figure 2 present the results from the mixed effects models and effect sizes for the continuous outcomes.

#### Primary Outcome

For PCL-C, a model with a quadratic term, indicating a nonlinear development over time, provided best fit to the data (see Multimedia Appendix 1). The intervention group exhibited a significant decline that abated over time. The control group showed no change. The model difference at postassessment was 9.16 points on the PCL-C in favor of the intervention group, representing a large effects size. At follow-up, the model difference was 8.07 points on the PCL-C in favor of the intervention group, representing a medium to large effect size.

#### Secondary Outcomes

For BDI-II, a linear model provided best fit to the data (see Multimedia Appendix 2). The intervention group exhibited a significant decline over time. The control group showed no change. The model difference at postassessment was 3.91 points on the BDI-II in favor of the intervention group, representing a medium effect size. The model difference at follow-up was 9.41 points on the BDI-II, representing a large effect size. For BAI, a linear model provided best fit to the data (see Multimedia Appendix 3). At preassessment, the intervention group reported a significantly higher level of symptoms; the model difference was 5.11 points on the BAI. The intervention group exhibited a significant decline over time, whereas the control group exhibited a significant increase in symptoms over time. At postassessment, there was no difference between the groups. However, at follow-up there was a difference of 6.99 points on the BAI in favor of the intervention group, representing a large effect size

Results for the economic variables are presented in Multimedia Appendix 4. Significantly more parents in the intervention group had seen a social worker at preassessment. No other differences between the groups were evident.

## Discussion

To the best of our knowledge, this is the first study to report on the long-term outcomes of guided self-help via the Internet for parents of children on cancer treatment. The results indicate that the intervention was effective in terms of reductions in the primary outcome PTSS and that these improvements were maintained at the 12-month follow-up. Significant reductions for the intervention group compared with the control group were also evident for the secondary outcomes of depression and anxiety, and between-group effect sizes were even larger at the 12-month follow-up. However, there was no support for the intervention being effective in reducing health care consumption or sick leave.

The findings that psychological distress can be reduced among parents of children with cancer are in line with previous investigations on problem-solving training administered in face-to-face format for mothers [11,12]. In the trial of problem-solving training compared with an inactive control condition [11], similar standardized mean differences between groups, as outlined in this study as metrics of effect-sizes, were not presented, which makes it hard to compare the treatment effects of these two trials. However, in the problem solving trial, significant intervention effects were observed for PTSS and depression at the end of the intervention (3 months post randomization), and these differences were maintained at the follow-up, 6 months post randomization. The results presented herein extend these findings and suggest that significant intervention effects for PTSS and depression observed post intervention can be maintained (PTSS) and seemingly improved (depression) at 12-month follow-up. In addition to mode of administration, another important difference between the two trials is that the current trial included participants reporting an increased level of PTSS, whereas the problem-solving trial included mothers irrespective of self-reported level of distress. Arguably, this results in different populations, which suggest that comparisons between the trials should be made with caution.

We did not find support for treatment efficacy in terms of reduced health care consumption or sick leave. Importantly, the small sample size and substantial attrition made it difficult to use estimation techniques that are in line with the ITT principle (eg, general estimating equations), and we were forced to base the analysis on completers. Furthermore, the frequencies of some of the indicators for health care consumption and sick leave were small and with little variation. It is also important to note that previous research regarding economic strains on families caring for a child with cancer has mainly concerned working hours and expenses [5,6]. Such factors may well impact on parents level of distress as economic burden may be an additional stressor for the parents. However, psychological distress may not impact on working hours and expenses. In this study, we strived to assess economic factors that may be influenced by the parents’ level of distress, such as health care consumption and sick leave and to investigate whether reductions in psychological distress would be associated with reductions in health-related expenses or sick leave. Future research should aim to disentangle whether psychological distress contributes to strains on household economy among parents of children on cancer treatment. Such efforts should be able to better prepare research that aims to alleviate the economic burden imposed on parents on children with cancer, be it via psychosocial interventions or targeted policies. It may also be the case that we used a less than optimal instrument for assessing health-related consumption and sick leave. Future research is needed to develop reliable and valid instruments for assessing these factors in the current population, and such research could preferably start with qualitative methods in order to explore the phenomena of health-related costs in this population.

For the clinical outcomes, including the primary outcome PTSS, we used ITT-analyses with maximum likelihood estimation under the assumption of data missing at random, and under such assumption, this procedure produces less biased estimates compared with, for example, last-observation carried forward [31]. The results from these analyses indicate that the intervention produces substantial long-term improvements in PTSS, depression, and anxiety. However, using an inactive control group makes it impossible to draw conclusions regarding the specificity of the intervention. Future trials should include active control conditions that allow for the control of the nonspecific factors (eg, attention, information, and social support) that psychological interventions may be associated with. Furthermore, the sample was small, and attrition was substantial, which may limit the validity of the ITT-analyses and may hamper both the reliability and generalizability of the findings. In addition, the study is limited by the fact that reasons for not participating in the trial were not documented due to ethical reasons, which further limits the generalizability. As the study was small and underpowered, the risk for inflated effect sizes and type-I errors is also increased [34], which should be kept in mind when interpreting the findings. It is our impression that the intervention might have been too reliant on text and perhaps too time-consuming for parents in this difficult situation and might have deterred many from participating and also affected attrition. Potential adjustments include shortening the intervention and relying less on text in favor of other ways of communicating on the Web. In addition, fathers were less likely to participate at the 12-month follow-up than mothers, which may further limit the generalizability. Unfortunately, the small sample size made it difficult to further explore the underlying mechanism of this difference in attrition. Historically, fathers have been less involved in pediatric psychology research [35], and future research with parents of children with cancer should take steps to ensure involvement from parents of both sexes [36]. Finally, this study relied on self-reported outcomes, and participants were not blind to their study condition, which should be kept in mind when interpreting findings. This is a problem for all research on psychological interventions using inactive control conditions, and the fact that participants were aware of their study condition might have affected their expectancies for improvement differently. In this study, no such expectancies were assessed so we cannot estimate or control for this factor in the analyses. Nonspecific factors such as expectancy for improvement might contribute to improvements in all kinds of psychological interventions. An alternative design with an active comparison condition receiving some kind of support, and also assessing treatment credibility and expectancies for improvement in both conditions, would had enabled us to better control for these factors.

To the best of our knowledge, this paper presents the first trial of an intervention administered via the Internet aiming to reduce psychological distress among parents of children on cancer treatment. ITT-analyses indicate substantial improvements in PTSS, depression, and anxiety that are maintained or strengthened at 12-month follow-up. However, we found no evidence for effects on health care consumption or sick leave. As such, using the Internet to provide psychological interventions may be an effective mode of delivery for parents reporting an increased level of PTSS and who consider Internet-based interventions as a viable option. Future research should corroborate these findings and also develop and evaluate interventions and policies that may help ameliorate the economic burden that parents may face during their child´s treatment for cancer.

## Appendix

Multimedia Appendix 1 This table describes model development with increasing complexity for the outcome PCL-C. Model D was chosen as the best fitting model as indicated by the significant difference test of the −2loglikelhood statistic and the lowest BIC.

Multimedia Appendix 2 This table describes model development with increasing complexity for the outcome BDI-II. Model C was chosen as the best fitting model as indicated by the nonsignificant difference test of the -2loglikelhood statistic when comparing with model D and the lowest BIC.

Multimedia Appendix 3 This table describes model development with increasing complexity for the outcome BAI. Model C was chosen as the best fitting model because of the lowest BIC. When comparing model C with model D, the -2loglikelhood test was significant indicating a better model fit for model D; however, we chose to present results from the more parsimonious model C because of the lower BIC and because the quadratic*group growth factor was nonsignificant in model D.

Multimedia Appendix 4 Observed frequencies of health care consumption and sick leave.

Multimedia Appendix 5 CONSORT-EHEALTH checklist (V1.6.1).

## Abbreviations

AIC: Akaike information criteria
BAI: Beck Anxiety Inventory
BIC: Bayesian information criteria
BDI-II: Beck Depression Inventory-II
CBT: Cognitive behavior therapy
ITT: Intention to treat
PCL-C: PTSD-Checklist-Civilian version
