# Supplementary material for: Twelve-Month Follow-Up of a Randomized Controlled Trial of Internet-Based Guided Self-Help for Parents of Children on Cancer Treatment
Source: J Med Internet Res. 2017 Jul 27;19(7):e273. doi: 10.2196/jmir.6852 (PMC5553001; doi:10.2196/jmir.6852)
Supplement: Multimedia Appendix 1 [file jmir_v19i7e273_app1.pdf]

| PCL-C | | Model A | Model B | Model C | Model D |
|---|---|---|---|---|---|
| Nr of param. | | 3 | 5 | 7 | 9 |
| Fixed effects | | | | | |
| Intial stauts | Intercept | 45.27*** | 48.1*** | 46.04*** | 46.26*** |
| | | (1.39) | (1.45) | (2.14) | (2.09) |
| | Group | | | 3.83 | 5.22 |
| | | | | (2.29) | (2.86) |
| Rate of change | Linear | | -4.01** | -0.49 | -2.29 |
| | | | (1.10) | (1.37) | (4.06) |
| | Quadratic | | | | 1.11 |
| | | | | | (2.00) |
| | Linear*group | | | -6.95** | -21.87*** |
| | | | | (1.95) | (5.92) |
| | Quadratic*group | | | | 7.47* |
| | | | | | (2.90) |
| Variance components | | | | | |
| Level 1 | Within | 91.45* | 76.00* | 67.26** | 50.91* |
| | | (15.24) | (14.55) | (12.59) | (10;61) |
| Level 2 | Initial status | 64.03* | 53.62* | 63.89** | 67.46* |
| | | (21.37) | (19.48) | (20.14) | (19.11) |
| | Rate of change | | 7.05 | 1.30 | 6.36 |
| | | | (10.39) | (8.37) | (8.63) |
| Fit indices | | | | | |
| -2loglikelihood | | 985.65 | 970.49 | 959.13 | 944.46 |
| ΔD | | | 15.16*** | 11.36*** | 14.67*** |
| AIC | | 991.65 | 980.49 | 973.13 | 962.46 |
| BIC | | 1000.18 | 994.71 | 993.01 | 988.06 |

*Note.* ΔD, the test of the difference in -2loglikelihood statistic according to the chi-2 distribution. AIC, Akaike Information Criteria. BIC, Bayesian Information Criteria.

*P<.05

**P<.01

***P<.001
