# Supplementary material for: Twelve-Month Follow-Up of a Randomized Controlled Trial of Internet-Based Guided Self-Help for Parents of Children on Cancer Treatment
Source: J Med Internet Res. 2017 Jul 27;19(7):e273. doi: 10.2196/jmir.6852 (PMC5553001; doi:10.2196/jmir.6852)
Supplement: Multimedia Appendix 2 [file jmir_v19i7e273_app2.pdf]

BDI		Model A	Model B	Model C	Model D
Nr of param.		3	5	7	9
Fixed effects					
Intial stauts	Intercept	18.56***	20.22***	19.33***	19.29***
		(0.93)	(0.96)	(1.45)	(1.48)
	Group			1.61	2.35
				(1.99)	(2.02)
Rate of change	Linear		-2.30**	0.41	0.63
			(0.80)	(0.92)	(2.84)
	Quadratic				-0.09
					(1.40)
	Linear*group			-5.58***	-13.28**
				(1.31)	(4.13)
	Quadratic*group				3.90
					(2.03)
Variance components					
Level 1	Within	42;43*	32.61*	28.17*	25.00*
		(6.88)	(6.28)	(5.20)	(4.79)
Level 2	Initial status	28.55*	24.74*	31.98*	33.85*
		(9.18)	(8.76)	(9.36)	(9.37)
	Rate of change		6.93	1.52	2.01
			(5.29)	(3.65)	(3.48)
Fit indices					
-2loglikelhood		886.82	873.55	852.17	851.93
ΔD			13.27***	21.38***	0.24
AIC		892.82	883.55	872.17	869.93
BIC		901.35	897.77	892.08	895.53

Note. ΔD, the test of the difference in -2loglikelihood statistic according to the chi-2 distribution. AIC, Akaike Information Criteria. BIC, Bayesian Information Criteria.

*P<.05

**P<.01

***P<.001
