# Supplementary material for: Twelve-Month Follow-Up of a Randomized Controlled Trial of Internet-Based Guided Self-Help for Parents of Children on Cancer Treatment
Source: J Med Internet Res. 2017 Jul 27;19(7):e273. doi: 10.2196/jmir.6852 (PMC5553001; doi:10.2196/jmir.6852)
Supplement: Multimedia Appendix 3 [file jmir_v19i7e273_app3.pdf]

BAI		Model A	Model B	Model C	Model D
Nr of parameters		3	5	7	9
Fixed effects					
Intial stauts	Intercept	13.36***	14.20***	11.48***	11.89***
		(0.97)	(1.02)	(1.52)	(1.52)
	Group			5.11*	5.27*
				(2.08)	(2.08)
Rate of change	Linear		-1.22	1.84*	-1.81
			(0.74)	(0.83)	(2.73)
	Quadratic				1.90
					(1.35)
	Linear*group			-6.06***	-8.57*
				(1.17)	(3.90)
	Quadratic*group				1.23
					(1.91)
Variance components					
Level 1	Within	35.1*	30.48*	22.93*	21.1*
		(6.04)	(6.23)	(4.53)	(4.11)
Level 2	Initial status	35.85*	32.97*	40.65*	41.20*
		(10.65)	(10.34)	(11.06)	(10.80)
	Rate of change		4.39	0.23	0.96
			(4.58)	(3.04)	(2.97)
Fit indices					
2loglikelhood		864.01	859.48	837.70	830.83
ΔD			4.53	21.78***	6.87*
AIC		870.01	869.48	851.7	848.83
BIC		878.50	883.62	871.5	874.29

*Note.*ΔD, the test of the difference in -2loglikelihood statistic according to the chi-2 distribution. AIC, Akaike Information Criteria. BIC, Bayesian Information Criteria.

*P<.05

**P<.01

***P<.001
