# Supplementary material for: Twelve-Month Follow-Up of a Randomized Controlled Trial of Internet-Based Guided Self-Help for Parents of Children on Cancer Treatment
Source: J Med Internet Res. 2017 Jul 27;19(7):e273. doi: 10.2196/jmir.6852 (PMC5553001; doi:10.2196/jmir.6852)
Supplement: Multimedia Appendix 4 [file jmir_v19i7e273_app4.pdf]

| | | Intervention | Control | Chi-square[a] | | *P* value |
|---|---|---|---|---|---|---|
| Pre | | (n=31) | (n=27) | | | |
| **Health care contact during the last month, n (%)** | | | | | | |
| | GP[b] | 8 (26) | 3 (11) | 2.03 | | .15 |
| | Occupational health | 1 (3) | 0 (0) | | | >.99 |
| | Social worker | 20 (65) | 8 (30) | 7.03 | | .01 |
| | Physiotherapist | 4 (13) | 2 (7) | | | .68 |
| | Mental health, private practice[c] | 3 (10) | 2 (7) | | | >.99 |
| | Mental health, community outpatient[d] | 2 (7) | 0 (0) | | | .49 |
| | Specialist physician, general hospital | 5 (16) | 3 (11) | | | .71 |
| | Specialist physician, university hospital | 3 (10) | 4 (15) | | | .69 |
| | Sick leave during the last month, yes versus. no | 8 (27) | 4 (15) | 1.20 | | .27 |
| Post | | (n=17) | (n=16[d]) | | | |
| **Health care contact during the last month, n (%)** | | | | | | |
| | GP | 6 (35) | 2 (13) | | | .23 |
| | Occupational health | 0 (0) | 0 (0) | | | - |
| | Social worker | 5 (29) | 5 (31) | | | >.99 |
| | Physiotherapist | 0 (0) | 0 (0) | | | - |
| | Mental health (private practice)[c] | 1 (6) | 1 (6) | | | >.99 |
| | Mental health (community outpatient)[d] | 0 (0) | 3 (19) | | | >.99 |
| | Specialist physician (general hospital) | 1 (6) | 0 (0) | | | >.99 |
| | Specialist physician (university hospital) | 0 (0) | 2 (13) | | | .23 |
| | Sick leave during the last month, yes versus no | 5 (31) | 2 (13) | | | .39 |
| 12-month follow-up | | (n=15[f]) | (n=16) | | | |
| **Health care contact during the last month, n (%)** | | | | | | |
| | GP | 4 (27) | 2 (13) | | | .39 |
| | Occupational health | 1 (7) | 0 (0) | | | .48 |
| | Social worker | 4 (27) | 4 (25) | | | >.99 |
| | Physiotherapist | 3 (20) | 1 (6) | | | .33 |
| | Mental health, private | 0 (0) | 2 (13) | | | .48 |

| | | | | | | |
|---|---|---|---|---|---|---|
| | practice[c] | | | | | |
| | Mental health, community outpatient[d] | 0 (0) | 0 (0) | | | - |
| | Specialist physician, general hospital | 2 (13) | 0 (0) | | | .23 |
| | Specialist physician, university hospital | 2 (13) | 2 (13) | | | >.99 |
| | Sick leave during the last month, yes versus no | 3 (20) | 2 (13) | | | .65 |

[a]Chi-square: *P* values reported are for chi-square tests (or Fisher exact test when no statistic is provided) with two-sided probabilities.

[b]GP: general practitioner.

[c]Includes psychologist, psychiatrist, and psychotherapist in private practice.

[d]Includes psychologist, psychiatrist, and psychotherapist in community outpatient settings.

[e]Four participants who participated in the postassessment had missing data on health care consumption and sick leave.

[f]One participant who participated in the postassessment had missing data on health care consumption and sick leave.
